# Evaluation and intention to use the interactive robotic kitchen system AuRorA in older adults

**DOI:** 10.1007/s00391-022-02105-8

**Published:** 2022-08-25

**Authors:** Luis Perotti, Nicole Strutz

**Affiliations:** Department of Geriatrics and Medical Gerontology, Charité — Universitätsmedizin Berlin, corporate member of Freie Universität Berlin and Humboldt-Universität zu Berlin, Reinickendorfer Str. 61, 13347 Berlin, Germany

**Keywords:** Assistive technology, Elderly, Meal preparation, Usability, Ageing in place, Assistive Technologie, Ältere Menschen, Nahrungszubereitung, Benutzerfreundlichkeit, Selbstständigkeit

## Abstract

**Background:**

The number of older adults in need of care and living at home is increasing in Europe. At the same time, the number of professional caregivers is decreasing. This development reinforces the need for assistive technology to support care recipients in their own homes and promote their independence. One of the main challenges of independent living is the preparation of food. Interactive robots could assist older adults with difficulties performing physically demanding tasks. Within the project AuRorA (full German project title: Wiederverwendbare, interaktive Verhalten für proaktive Roboter im Smart Home), an interactive voice-controlled robot arm was developed as an assistance system in the kitchen.

**Objective:**

The aim of the study was to assess how older adults evaluate the AuRorA system and to collect data on actual willingness to use the technology. Older adults were asked to evaluate the system in terms of usefulness, usability, accessibility and intention to use.

**Material and methods:**

Due to the ongoing coronavirus disease 2019 (COVID-19) pandemic, the older adults evaluated the system via an online survey. The validated questionnaire Technology Usage Inventory (short: TUI) was used in conjunction with self-developed questionnaires to collect data on study population characteristics.

**Results:**

A total of 106 participants were included in the analysis. The acceptance, usability and usefulness of the system were rated as medium, while the intention to use was rated as low. A significant strong correlation was found between the TUI subscales intention to use and usefulness.

**Conclusion:**

It can be assumed that the actual need of the individual participant for such a robotic assistive system had an influence on the evaluation of the system. The perceived usefulness may have been a crucial influence on the intention to use and the overall assessment of the system.

**Supplementary Information:**

The online version of this article (10.1007/s00391-022-02105-8) contains supplementary material, which is available to authorized users.

## Abstract (de)

**Hintergrund:**

Die Zahl älterer pflegebedürftiger Menschen, die in der eigenen Häuslichkeit leben, nimmt in Europa stetig zu. Gleichzeitig nimmt die Zahl der professionell Pflegenden ab. Assistive Technologien könnten diese Menschen entlasten und ihre Unabhängigkeit fördern. Eine der größten Herausforderungen eines unabhängigen Lebens ist die Zubereitung von Speisen. Bei der Ausführung solch körperlich herausfordernder Tätigkeiten könnten Assistenzroboter unterstützen. Im Rahmen des Projekts AuRorA (vollständiger Projekttitel: Wiederverwendbare, interaktive Verhalten für proaktive Roboter im Smart Home) wurde ein interaktiver, sprachgesteuerter Roboterarm für den Einsatz in der Küche entwickelt.

**Ziel der Arbeit:**

Ziel der Studie war es, festzustellen, wie ältere Menschen das AuRorA-System bewerten, und die tatsächliche Bereitschaft zur Nutzung der Technologie zu erheben. Das System wurde von im Hinblick auf Nützlichkeit, Benutzerfreundlichkeit, Zugänglichkeit und Nutzungsabsicht bewertet.

**Material und Methoden:**

Aufgrund der andauernden COVID-19-Pandemie wurde die Evaluation des Systems durch ältere Menschen mittels Online-Befragung durchgeführt. Der validierte Fragebogen Technology Usage Inventory (kurz: TUI) wurde gemeinsam mit selbstentwickelten Fragebögen zur Erhebung von Basisdaten verwendet.

**Ergebnisse:**

Insgesamt wurden 106 Teilnehmer*innen in die Analyse einbezogen. Die Akzeptanz, Benutzerfreundlichkeit und Nützlichkeit des Systems wurden als mittelhoch eingestuft, während die tatsächliche Nutzungsabsicht als gering eingestuft wurde. Es wurde eine signifikante starke Korrelation zwischen den Subskalen des TUI Nutzungsabsicht und Nützlichkeit festgestellt.

**Diskussion:**

Es kann davon ausgegangen werden, dass der tatsächliche Bedarf der einzelnen Befragten an dieser Art robotischen Assistenzsystems einen Einfluss auf die Bewertung hatte. Es wird ebenso vermutet, dass die wahrgenommene Nützlichkeit einen entscheidenden Einfluss auf die Nutzungsabsicht und die Gesamtbewertung des Systems hatte.

## Introduction

The majority of older adults in need of care are cared for in their own homes by professional or informal caregivers. The preparation of meals in particular is difficult for people with physical disabilities. Assistive robotic technologies could relieve the burden on caregivers and increase the independence of users; however, there is little research on how older adults evaluate such technologies for home use and on their actual willingness to use them.

## Background

### The living and care situation of older adults

The continuing growth of the proportion of older adults in European has been accompanied by an increase in the number of people in need of long-term care. At the same time, most older adults wish to remain in their own living situation for as long as possible and without the support of others. Even in the event of them needing care, they often prefer to be cared for at home by outpatient care services [12]. Remaining in a familiar living situation has a proven positive effect on the life satisfaction of those affected [19]. Older adults in need of care particularly need support for more complex activities associated with daily living (called instrumental activities of daily living), such as going shopping or preparing meals [3]. At the same time, however, there is also a persistent shortage of nursing staff in Germany providing home care [13].

These changes in society and the concomitant increased need for home care strengthen the demand for technological assistance systems to enhance the quality of life, health and independence of those in need of care and to relieve the burden on caregivers. The positive effects on health and quality of life resulting from integrating modern technologies into the daily lives of older adults are well documented [23].

Klein refers to eating and drinking as “basic activities” that play a central role in every life [15]. However, most assistive systems available in Germany only serve the purpose of supporting the intake of food, but do not offer help preparing it [21]. The kitchen is seen as a central area where older adults perceive the greatest need for support from assistance systems [9].

Against the background of this problem, the AuRorA project was initiated (full German project title: Wiederverwendbare, interaktive Verhalten für proaktive Roboter im Smart Home), which aims to support older adults in preparing food in their own homes by developing an innovative interactive robotic solution.

### The AuRorA system

**Fig. 1:**
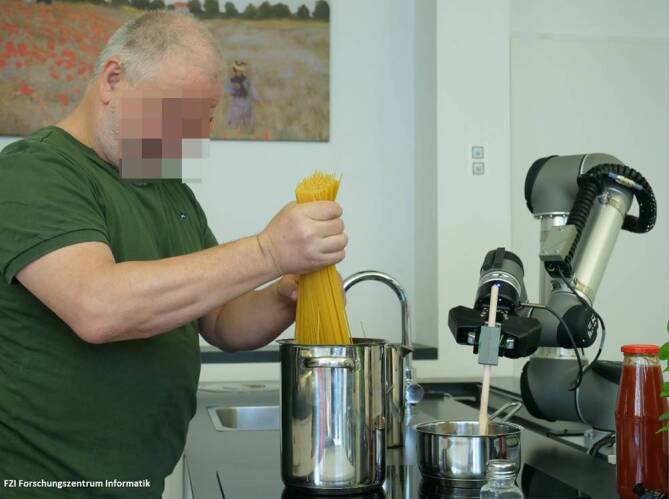
The AuRorA (full German project title: Wiederverwendbare, interaktive Verhalten für proaktive Roboter im Smart Home) system in use (Source: permission for use granted by FZI Forschungszentrum Informatik)

At the core of the assistance system AuRorA is the lightweight robotic arm Universal-Robot UR5 (Universal Robots, Odense, Denmark) (Fig. 1). The robotic arm can grasp objects with the help of its two-fingered grasping device. According to the systematization of robots for use in healthcare by Kehl, AuRorA can be classified as a robot for personal assistance in everyday tasks [14]. The system is controlled via voice control and designed to be as cooperative and work-sharing as possible in order to involve the user in the activity being carried out. A participatory research and development approach was adopted within the project. Feedback from the target group was collected in several evaluations and taken into account. The level of participation can be rated as medium according to Merkel and Kucharski [18].

### Research questions

De Graaf et al. stated that in the evaluation of a robotic technology for personal and domestic use, specific emphasis should be placed on exploring the target group’s acceptance and intention to use the technology. Acceptance of robotic technologies by older adults has been found to be closely related to the perceived usefulness of these systems [7]; however, perceived usability was also identified as a key factor for older adults when evaluating a system [11]. These aspects, as well as the resulting willingness of older adults to use the AuRorA system, are the subjects of the study presented here.

We sought to answer the following research questions:

1. What are the basic attitudes of older adults towards the technology presented in this study?
2. How do older adults evaluate the AuRorA system in terms of accessibility, perceived usability and usefulness?
3. How do older adults evaluate the intention to use the robotic assistance system?

## Study design and investigation methods

**Fig. 2:**
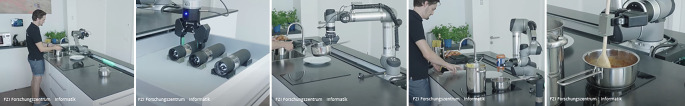
Screenshots of the video used in the survey, (Source: permission for use granted by FZI Forschungszentrum Informatik)

Due to the ongoing coronavirus disease 2019 (COVID-19) pandemic, we conducted an online survey so that the study could be carried out without direct face-to-face contact. We created a detailed video of a prototypical cooking process with the AuRorA system to be used in the survey (Fig. 2). We clearly presented the workflow of the interactive cooking process. We showed the resulting video to a number of representatives of the target group before the start of the study and checked it for comprehensibility.

We used the following questionnaires within the survey:

### Basic data questionnaire

We collected basic data about the person completing the questionnaire. This information included the subject’s age, sex, technology usage and officially recognized degree of disability.

### Technology Usage Inventory

The validated Technology Usage Inventory (short: TUI) was used for the evaluation of the AuRorA system [16]. The answers for the scales curiosity, technology anxiety, interest, usability, usefulness, skepticism and accessibility are given on a 7-point Likert scale (1 = “strongly disagree”, 7 = “strongly agree”). Answers about respondents’ intention to use the system are given on a visual analog scale (maxima: 0 = “agree”, 100 = “disagree”). Due to the orientation of the other scales of the TUI, we reversed the intention to use subscale in the results section of this paper.

### Survey design and distribution

For the development of the digital assessment underlying this study, we used the tool REDCap (Vanderbilt University, Nashville, TN, USA) [10].

We sent the survey to the members of the internal database of the Geriatrics Research Group of Charité — Universitätsmedizin Berlin via email. We also contacted various senior citizen facilities that agreed to forward the link. Healthy individuals with a minimum age of 65 years and internet access were included in the study.

### Data analysis

We analyzed the data using IBM SPSS Statistics 27 (IBM, Armonk, NY, USA). For all the variables, we calculated mean values and standard deviations. Since ordinal data were present, we calculated the Spearman’s rank correlation coefficient to measure the correlation between the key variables of the TUI. We specified a significance level of 0.05 for the analysis. To interpret the calculated Spearman’s rank correlation coefficients, we considered the approach and cut-off values by Schober et al. [22].

## Results

### Study sample characteristics

Table 1 shows sociodemographic data as well as further data about the study participants’ characteristics.

**Table 1:** Study population characteristics

Study population characteristics, *n *(total) = 106	
*Age (mean value)*	73.78 years (SD = 5.138)
*Sex (n* *=* *4 missing)*	
Male	30.39%
Female	69.61%
*Officially recognized degree of disability (n* *=* *2 missing)*	
No	70.19%
Yes	29.81%
*Technology usage (n* *=* *1 missing)*	
Never or rarely	5.8%
Occasionally	13.3%
Often	81%

*SD* standard deviation

### Results of the TUI

#### Evaluation of the TUI and its subscales

The results of the TUI questionnaire are presented in Table 2.

**Table 2:** Results of the Technology Usage Inventory (TUI)

Scales	Results *n* = 106
Usability (min.: 3, max.: 21), *n* = 16 missing	14.93 (SD = 3.95)
Accessibility (min.: 3, max.: 21), *n* = 16 missing	7.29 (SD = 3.46)
Curiosity (min.: 4, max.: 28), *n* = 8 missing	16.01 (SD = 6.21)
Technology anxiety (min.: 4, max.: 28), *n* = 7 missing	10.55 (SD = 5.45)
Skepticism (min.: 4, max.: 28), *n* = 17 missing	14.61 (SD = 5.18)
Usefulness (min.: 4, max.: 28), *n* = 15 missing	10.31 (SD = 5.83)
Interest (min.: 4, max.: 28), *n* = 15 missing	16.57 (SD = 6.13)
Intention to use (scale reversed) (min.: 3, max.: 300), *n* = 17 missing	80.19 (SD = 76.51)

*min* scale minimum, *max* scale maximum, *SD* standard deviation

### How do older adults evaluate the AuRorA system in terms of accessibility, perceived usability and usefulness?

The average score for accessibility of the AuRorA system was 7.29 (SD = 3.46). A look at the individual items shows that financial accessibility in particular (“I think almost everyone can afford this technology.”) was rated critically (mean score = 1.95, SD = 1.22). Similarly, evaluation of the acquisition effort involved (“I think that acquiring this technology does not require much effort.”) achieved a low score of 2.29 (SD = 1.65).

**Fig. 3:**
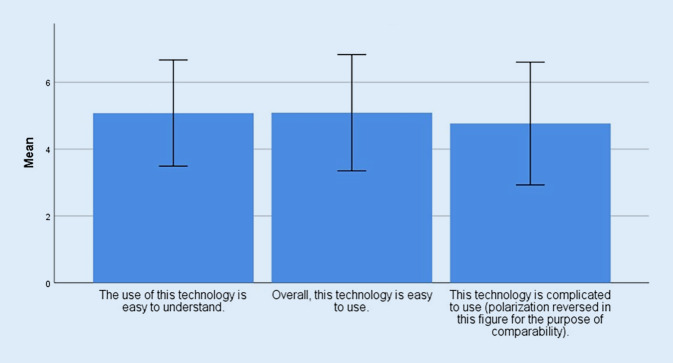
Mean values TUI (Technology Usage Inventory) subscale “usability” with standard deviation (SD)

The perceived usability of the assistance system achieved a mean score of 14.93 (SD = 3.95), placing it just in the upper third of the scale. Likewise, the scores for individual items showed that application and comprehensibility of the system’s operation were rated rather positively (Fig. 3).

**Fig. 4:**
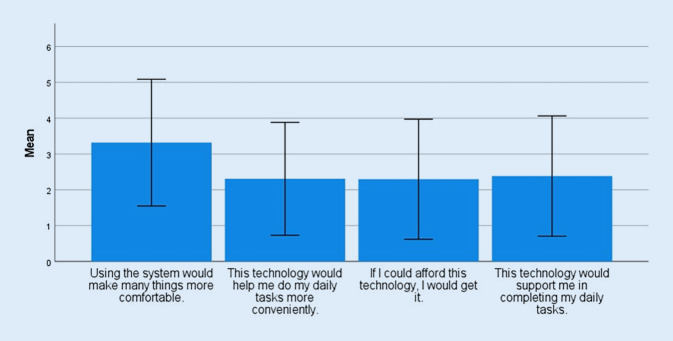
Mean values TUI (Technology Usage Inventory) subscale “usefulness” with standard deviation (SD)

With respect to the usefulness subscale, study participants’ evaluation of the system resulted in a mean overall score of 10.31 (SD = 5.83), which corresponds to a value in the lower third of the scale. Here, a look at the underlying values of individual questions is particularly informative (Fig. 4). The first question refers to evaluation of the expected gain in comfort from using the system. This item achieved a mean score of 3.35 (SD = 1.75). In contrast, the ability of the system to provide support in completing daily tasks was rated with a mean score of 2.43 (SD = 1.74).

### How do older adults evaluate the intention to use the robotic assistance system?

**Fig. 5:**
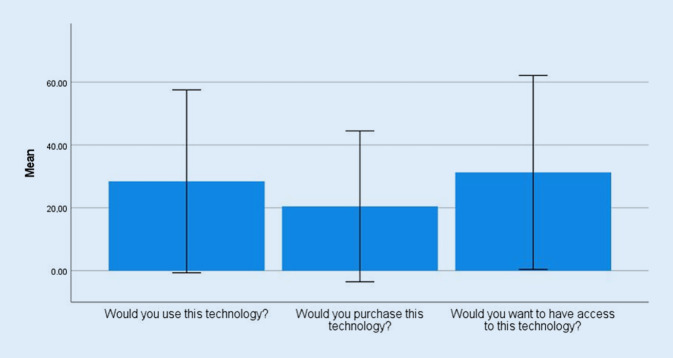
Mean values TUI (Technology Usage Inventory) subscale “intention to use” with standard deviation (SD)

The total score given by the older adults surveyed in this study in relation to their intention to use the system was 80.19 (SD = 76.51). This is in the lower third of the overall scale. The intention to use scale consists of three questions, concerning respondents’ actual intention to use, intention to purchase and desire to have access to the technology. The mean scores for all three questions reached the lower third of the scale (Fig. 5). The intention to purchase reached a mean score of 21.22 (SD = 24.09) and the desire to access it a mean score of 31.28 (SD = 30.85).

For a better understanding of factors which may have an influence the intention to use by the older adults, we calculated correlation coefficients between the intention to use and other factors (Table 3). The interpretation of the Spearman’s rank correlation coefficients shows that the greatest correlation is found between the subscales intention to use and usefulness. A strong correlation is present between the variables (Spearman’s ρ = 0.742, *p* < 0.001) with greater perceived usefulness being related to higher intention to use [22]. Other significant correlations exist between the intention to use subscale and the curiosity and skepticism subscale. However, only the correlation between intention to use and curiosity (Spearman’s ρ = 0.414, *p* < 0.001) as well as the intention to use and skepticism (Spearman’s ρ = −0.45, *p* < 0.001) are in the range of a moderate correlation [22]. It can be seen that higher skepticism is related to lower and higher curiosity is related to higher intention to use.

**Table 3:** Spearman’s Rho: correlation between variables and intention to use (ITU)

Variable	Curiosity	Skepticism	Usability	Usefulness	Accessibility	Sex	Degree of disability (presence)	Technology usage (occasionally/frequently)
Correlation coefficient	0.414	−0.45	0.095	0.742	0.171	−0.137	0.085	0.057
Sig. (2-tailed)	0.000*	0.000*	0.389	0.000*	0.112	0.207	0.432	0.595
*N*	86	84	85	86	88	87	88	89

*ITU* intention to use, *Sig.* statistical significance

*p < 0.05

## Discussion

The medium to low evaluation of the AuRorA system in this study is reflected by the results of other studies that have researched the acceptance of older adults regarding robotic assistance systems. Many studies reported a low level of willingness to use and implement robotic solutions for the target group [2, 20]. Bronswijk et al. have pointed out that acceptance of ambient assisted living technology is a key hurdle in implementation of such systems in the lives of older adults due to the technology being perceived as unnecessarily complicated [5]. Contrary to the results from these studies, the participants of the present study evaluated the perceived usability of the AuRorA system rather positively.

The number of people with an officially recognized degree of disability was relatively small in this study. The medium to low ratings for acceptability and usefulness of the AuRorA system could be due to the lack of the individual need for assistance among the study participants. Heerink et al. described the perceived usefulness as a crucial factor influencing the intention to use an assistance system [11]. This goes in line with the results of the study presented here in which a strong correlation between the rating of the usefulness and the intention to use was present.

The eighth Ageing Report of the German Government emphasized the importance of taking ethical considerations into account when exploring the development, distribution and usage of digital technologies for older adults [4].

In this context, ethical aspects that are important regarding presented technology should be discussed. First, the aspect of distributive justice needs to be addressed. The AuRorA kitchen system is a functional demonstrator that is not yet ready for introduction into the market. However, it is foreseeable that both acquisition and installation will be very costly. Within this study, the participants critically assessed the affordability of the system. The medium rating for accessibility of the system in this study (particularly evident in relation to the financial accessibility subscale) is in line with the results of other studies. Becker et al. have also emphasized the concern of older adults that due to the unclear financing possibilities of robotics for home use, they are uncertain about the financial feasibility of such a system [1]. Especially older adults with a lower socioeconomic background show a higher prevalence of need for care, while having fewer resources for financing expensive assistive technology [17]. At the same time, there is a possibility that a perceived stereotyping of study participants as potentially in need of assistance influenced the evaluation of the system. Flandorfer speaks of the danger of stereotyping older adults when developing and evaluating new technologies [6]. A more specific definition of the target group for this study (older adults with food preparation support needs) could have counteracted this.

The low ratings for the perceived usefulness of the AuRorA system may be influenced by the general attitude of older adults towards robotic systems [20]. This assumption is supported by the significant moderate correlation between the intention to use and the skepticism and curiosity subscales. It indicates that the individual attitudes and the preconceptions towards the robot influenced the intention to use the system. Graf noted that the acceptance of a robot with caregiving tasks is highly dependent on the individually perceived fields of application and the amount of abilities of the robot. He suggested that older adults wish to use assistive technology in basic in the handling of heavy objects, but for some activities, such as food preparation, they prefer human assistance [8]. The wish for human interaction during meal preparation may have influenced the assessment of the system’s usefulness.

In this study, only an officially recognized degree of disability was queried, which, however, does not necessarily have to imply a limitation in the preparation of food. Thus, as a further evaluation step studies should enroll more participants with support needs in relation to food preparation. Furthermore, the specific needs and technical competencies of especially vulnerable groups, such as people with dementia, should be addressed in the future development of the AuRorA system.

## Limitations

- Less tech-savvy individuals may not have been reached by the online survey. Survey methods like face-to-face interviews are preferable for reaching the target group.
- Mostly healthy older adults were included in this study. The results cannot be generalized to older adults in the need of support for preparing meals.

## Conclusion

- There are still barriers to introduction of assistive robotics for older adults, especially financial barriers were considered critically.
- The perceived usefulness may have a crucial influence on the intention to use and the overall evaluation of the system.

### Supplementary Information

The AuRorA robotic system

Technology commitment of the study participants

## Declarations

All procedures performed in studies involving human participants or on human tissue were in accordance with the ethical standards of the institutional and/or national research committee and with the 1975 Helsinki declaration and its later amendments or comparable ethical standards. Informed consent was obtained from all individual participants included in the study. The study was approved by the Ethics Committee of the Charité — Universitätsmedizin Berlin (EA4/101/20) prior to commencement.
