# Supplementary material for: Evaluation and intention to use the interactive robotic kitchen system AuRorA in older adults
Source: Z Gerontol Geriatr. 2022 Aug 25;56(7):580–6. doi: 10.1007/s00391-022-02105-8 (PMC9406253; doi:10.1007/s00391-022-02105-8)
Supplement: Supplementary file 1 — The AuRorA robotic system [file 391_2022_2105_MOESM1_ESM.docx]

**Supplementary material – The AuRorA robotic system**

**The AuRorA system: Description**

At the core of the assistance system developed in this project is a lightweight robotic arm, known as the “Universal-Robot UR5”. The robotic arm was developed to support people in need of care in their own homes by enabling physical manipulation of objects in the context of a cooking process via voice command. In order to classify the system in the field of already existing systems of assistive robotics, Kehl's systematization can be used. In distinction to devices assisting mobility and robots for social and emotional support, Kehl speaks of robots for physical assistance in everyday tasks [2]. In this context, Becker speaks of robots for personal assistance that increase the user's independence in everyday life [1]. The AuRorA system matches these approaches to systematization. The robotic arm has 6 degrees of freedom in its movements and can grasp objects with the help of its two-fingered grasping device. This function is supported by an object recognition software application, developed as part of the project. Object recognition allows the robot arm to avoid obstacles within its trajectory. In addition, the arm can also avoid the human user if the user unexpectedly enters the range of action of the robot arm. The robot arm is embedded in a smart kitchen, which has automatically extendable drawers and automatic stovetops (see Figure 1). The system is controlled via voice control, whose dialog tree was implemented during the project. Part of the innovative approach of the system is the control by voice in combination with the collaborative approach to the cooking process. The assistance system is designed to be as cooperative and work-sharing as possible in order to involve the user in the activity being carried out. This means that the system can independently perform certain tasks, but needs the human user to carry out the full cooking process. On the one hand, this is intended to maintain the user’s level of activity within the scope of his or her cognitive and physical capabilities and, on the other hand, promote human-robot interaction.

The development process was carried out with close involvement of the target group of older adults. Iterative testing and interviews with older adults, as well as demonstrations and testing of the voice control, took place. Feedback was obtained in all project phases (by means of qualitative and quantitative data collection) and was taken into account in further development steps. To ensure the safety of users, comprehensive risk management was carried out during development. This involved evaluating all potential risks for users and responding appropriately to minimize these. For example, the maximum movement speed of the arm was reduced.

1. Becker H, Mandy S, Michael F et al (2013) Robotik in Betreuung und Gesundheitsversorgung. :227 S. https://doi.org/10.3929/ETHZ-A-007584670

2. Kehl C (2018) Robotik und assistive Neurotechnologien in der Pflege - gesellschaftliche Herausforderungen. Vertiefung des Projekts »Mensch-Maschine-Entgrenzungen«. https://doi.org/10.5445/IR/1000094095
