# Supplementary material for: Evaluation and intention to use the interactive robotic kitchen system AuRorA in older adults
Source: Z Gerontol Geriatr. 2022 Aug 25;56(7):580–6. doi: 10.1007/s00391-022-02105-8 (PMC9406253; doi:10.1007/s00391-022-02105-8)
Supplement: Supplementary file 2 — Technology commitment of the study participants [file 391_2022_2105_MOESM2_ESM.docx]

**Supplementary material – Technology commitment of the study participants**

**Supplement to: Study design and investigation methods**

*Questionnaire on technology commitment*

Using the questionnaire on technology commitment developed and validated by Neyer et al., the attitudes and dealings of the target group in relation to modern technology were surveyed [1]. The questionnaire was divided into various subscales: technology acceptance, technology control conviction and technology competence conviction. The questionnaire included 12 questions and could be answered with a five-point Likert scale (ranging from 1=“not at all true” to 5=“completely true”).

**Supplement to: *Study sample characteristics***

*Results of the technology commitment assessment*

The individuals who participated in the study achieved a total score of 3.55 (SD=0.61) for technology commitment across all items in the survey instrument. This score is slightly below the mean technology commitment score of the validation study sample for the instrument (Neyer et al., 2016). In this, individuals from all age groups over 18 were surveyed, reaching a mean score of 3.71 (SD=0.62). The scores for individual subscales of the instrument were also slightly lower than those of the validation study (see Table 1).

*Table 1. Results of the technology commitment assessment*

| **Scales** | **Present Study**  (mean age X̅=73.78 years, n=99) | **Validation Study**  (mean age X̅=40.6 years, n=825) |
| --- | --- | --- |
| Technology commitment – total score | 3.55 (SD=0.61) | 3.71 (SD=0.62) |
| Technology acceptance | 3.19 (SD=0.86) | 3.27 (SD=0.94) |
| Technology literacy beliefs | 3.94 (SD=0.95) | 4.16 (SD=0.8) |
| Technology control beliefs | 3.54 (SD=0.86) | 3.75 (SD=0.74) |

1. Neyer FJ, Felber J, Gebhardt C (2016) Kurzskala Technikbereitschaft (TB, technology commitment). Zusammenstellung Sozialwissenschaftlicher Items Skalen ZIS. https://doi.org/10.6102/ZIS244
